# Diphtheria in Bristol, 1921
*A Report to the Bristol Health Committee.


**Published:** 1922-02

**Authors:** D. S. Davies

**Affiliations:** (Medical Officer of Health)


### DIPHTHERIA IN BRISTOL, 1921.*

By D. S. DAVIES, M.D., D.P.H. (Medical Officer of Health).

THE disease appears to be of respectable antiquity, though it is difficult to identify every form of recorded angina. In 1389, however, a disease, probably diphtheria, carried off a large number of children in England. Between 1745 and 1750 outbreaks occurred in Cornwall, and, later in the century in London. At the end of the eighteenth century diphtheria, though still existent, no longer prevailed in epidemic form, though it persisted meanwhile in France, and in 1859 reappeared in the South of England, probably introduced from the Continent, and spread widely and fatally.

As diphtheria was, previously to the year 1855, not separated in the Registrar-General's death returns from scarlet fever, accurate statistical evidence is not obtainable, but from 1855 onwards the returns are of considerable interest. The abrupt rise, after a long period of quiescence, between the years 1857 and 1859, followed by an equally abrupt fall to a moderate average but one in excess of the quiescent period, is characteristic of the general behaviour of the disease, which observes cyclical periods of prevalence after irregular and somewhat prolonged intervals of quiescence.

During its prevalence from 1860 to the end of the century, diphtheria, at first favouring sparse localities and county districts, became more and more established as a town disease, and this change may be not entirely disconnected from the increasing regularity of school attendance under the provisions of the 1870 Education Act. While the age susceptibility to diphtheria is greatest at ages 1 to 5, the mortality between 5 and 10 years is still about double that at all ages, so that school attendance, bringing together as it does children at susceptible ages for many hours in close proximity with many opportunities for direct or indirect contact, assumes much importance in a disease which inhabits the mouth and nose cavities and depends for its spread upon transference of infective material from the sick to the healthy.

Diphtheria observes with much regularity a seasonal prevalence, having its highest period in the December quarter and its lowest period during the summer months, and the disease is commoner in temperate and cold climates than in tropical countries. It is highly probable that cold and wet influence the delicate mucous membrane towards susceptibility; the actual infection must, however, be received from an existing case.

The opinion that diphtheria is a filth disease, and that insanitary conditions are a direct cause, is no longer tenable; though such conditions may, no doubt, as may damp or inclement weather conditions, act as predisposing causes. The influence of the "carrier" condition, whether in a convalescent or in an apparently well person, was, until recently, imperfectly recognised, though Gresswell pointed out to the Epidemiological Society in 1885-1886 the possibility that diphtheria might persist after attack as a chronic malady. It is an interesting and important fact that diphtheria does not single out for attack the slum areas of a city, but may prevail in any district, rich or poor, where there is a sufficiency of children at susceptible ages who have opportunity of inter-communication.

Diphtheria did not become an urgent problem in Bristol until early in the nineties, neither did it make any call upon the hospital accommodation, nor had it become a serious difficulty in relation to school attendance. All this was changed by the wave of prevalence which, starting late in Bristol (about 1894), persisted over the end of the century, reached its highest point about the year 1902, and since that date, though less urgently prevalent, has given continuous anxiety.

The introduction of antitoxin about the year 1894 placed a powerful remedial measure, almost infallible if used early and boldly, in the hands of the physician, while the previous recognition of the casual organism by Klebs and Loeffler in 1883-1884, gave valuable aid, first in confirmation of clinical diagnosis, and secondly, in the searching out of contacts; and which, so long as bacteriological results are used to supplement and not to supplant clinical observation, and are not accepted as any excuse for delay in administering antitoxin, is also invaluable. The fatality of diphtheria, always serious, was favourably influenced by the introduction of antitoxin as a remedy, which still remains a powerful weapon, with the proviso that its value rapidly diminishes with each day's delay in its administration.

During the past year the prevalence in the district of a remarkably virulent type of diphtheria over which antitoxin, though given early and in full dose, failed to exert its wonted control, led at first to suspicion of the source of supply. This, however, proved groundless, and substituted antitoxin gave no better result. It was, therefore, assumed that the type of disease had changed towards greater virulence; and the effective dose is now found to be double or even treble that originally needed.

It is difficult to account fully for this accession of virulence, which probably depends on more than one factor; but it is worthy of note that Newsholme, as long ago as 1898, propounded a thesis that a succession of dry years tended to be accompanied or followed by a heightened prevalence and a more severe type of diphtheria. It is reasonable to suppose that, after such a continuance of dry seasons, the sensitive mucous membrane is more readily influenced towards susceptibility by cold or otherwise inclement weather.

The very serious outbreak of 1902 quite definitely established itself by a succession of school outbreaks, a result which has probably been averted during the present outbreak by the development and very complete organisation of the School Medical Service since the Education (Administrative Provisions) Act of 1907, and especially since the War; but the School Medical Service is not charged with the duty of dealing with infectious disease. The measures taken

* A Report to the Bristol Health Committee.

**Diphtheria in Bristol, 1921—(*cont.*).**

to deal with diphtheria in 1902 included the establishment in affected districts of several out-patient departments, where contacts harbouring the organism, but otherwise well, could, when singled out by laboratory observation, then under the immediate care of the Medical Officer in charge of the control work, be given appropriate treatment by a skilled nurse under direct medical supervision.

In the present outbreak, not only is school influence, although present in some degree, far less in evidence, but the scattered nature of the invasions would make it difficult to acquire convenient out-patient centres, if, indeed, any premises were likely to be available for the purpose; and the administrative medical duties of the Department have so widely increased, while the medical strength has remained at the 1886 level, that any work of this kind would be out of the question.

In consequence, any doubtful cases referred from the laboratory as reasonably " suspicious " have had to be accommodated in hospital, with the result that not only have the fever wards been fully occupied with diphtheria, to the exclusion of other disease in urgent need of isolation, but the cases have overflowed into the female sanatorium wards, which are no longer available for their legitimate purpose; and if the disease extends, the male wards may have to be similarly pressed into service. In 1902-3-4, Clift House was available, and was intermittently used in relief of Ham Green for diphtheria; this relief is no longer available and is greatly missed.

During the first three quarters of 1921 the deaths from diphtheria showed no remarkable excess above the mean, although in January a sharp outbreak in the wards of the Southmead Infirmary of the Bristol Union gave significant emphasis to th fact, already noted in the city hospitals, that a peculiarly virulent type of disease was present in the district.

The Southmead outbreak affected fifteen children, of whom five died, and 34 other cases, apparently of independent origin, and all followed by recovery, occurred at the Cottage Homes. Four members of the staff also experienced slight attacks.

The main importance of this Southmead outbreak is that it has given opportunity for acquiring a practical acquaintance with a procedure which, in the hands of observers in America, where diphtheria appears to be far more in evidence than in England, has proved highly satisfactory in protecting threatened groups or communities against diphtheria.

In order to ensure the adequate protection of the population, the New York system provides a medical service for carrying out the testing and immunising, especially amongst the school population. Briefly, the procedure is this :—

Upon the occurrence of a case of diphtheria in a school or institution :

1. All contacts are first of all *tested* (Schick reaction); this test shows which are susceptible or likely to take the disease, and which are insusceptible. This is determined in the course of three or four days.
2. All susceptibles are then *protected* by the proper administration of the protective agent—reinforced on two occasions at weekly intervals, when an active immunity is developed which is found to persist over at least 3½ years.

There appears to be undoubted evidence that persons giving a " negative " to the test are relatively immune to diphtheria. In a large home for infants where diphtheria had been endemic as late as 1915, the 37 per cent. susceptibles were duly protected, and for five years the institution has remained free from the disease (Copeman). The Schick test can also be applied with advantage in testing the patients, resident staff and nurses of infectious diseases hospitals, and in protecting those who are liable to contract the disease.

The necessity for immediately arranging for thus dealing with the City Hospital Staff is emphasised by the fact that within the last three months four members of the nursing staff at Ham Green have contracted diphtheria, while six members of the Novers Hill staff have also suffered non-fatal attacks, though no diphtheria was knowingly admitted to the wards. In the year 1920 no less than twelve members of the staff at Ham Green contracted diphtheria, one of whom died of a virulent attack, and in 1921 eleven further cases occurred at Ham Green. The duty of protecting a staff exposed to such serious risk is obvious, and the means are now available. It would be a lost opportunity were I not to contrast for your Committee's serious contemplation this terrible, sometimes fatal, and hitherto unpreventable morbidity amongst the staff engaged in nursing diphtheria with the complete immunity enjoyed by the staff engaged in nursing smallpox. We simply do not allow nurses to contract smallpox; there would seem to be no valid excuse now for permitting them to contract diphtheria.

The present outbreak commenced in October, 1921, and, though scattered, was at first notably in excess in certain streets in Bedminster. The neighbouring schools were proved not to be centres of infection, and as it appeared to be a " home " outbreak, every contact in these streets was searched out and tested for the presence of the diphtheria organism.

In October a second focus, this time a school, arose in Westbury, resulting in 23 cases and three deaths; the School Medical Officer co-operated in the necessary measures, a depot for the supply of antitoxin was arranged with a local chemist, contacts were dealt with and the outbreak subsided.

The third notable occurrence happened in this wise : a boy who was one of sixteen boarders at one house of a private school of 200 boys, went home for a week-end and slept with a brother who was at the time sickening for diphtheria. He returned to the boarding-house, and, himself sickening, infected two room-mates, and also two class-mates at school. Two boys died of these five attacked. As 200 boys were at risk, it was considered wise, from the experience already gained, to test, and if necessary protect, the remaining immediate contacts on the American method, and your Committee authorised this to be undertaken : no further cases have occurred.

